# Growth and Potential Damage of Human Bone-Derived Cells Cultured on Fresh and Aged C_60_/Ti Films

**DOI:** 10.1371/journal.pone.0123680

**Published:** 2015-04-15

**Authors:** Ivana Kopova, Vasily Lavrentiev, Jiri Vacik, Lucie Bacakova

**Affiliations:** 1 Institute of Physiology, Czech Academy of Sciences, Videnska 1083, 142 20, Prague, 4—Krc, Czech Republic; 2 Nuclear Physics Institute, Czech Academy of Sciences, 250 68, Rez near Prague, Czech Republic; University of Oulu, FINLAND

## Abstract

Thin films of binary C_60_/Ti composites, with various concentrations of Ti ranging from ~ 25% to ~ 70%, were deposited on microscopic glass coverslips and were tested for their potential use in bone tissue engineering as substrates for the adhesion and growth of bone cells. The novelty of this approach lies in the combination of Ti atoms (i.e., widely used biocompatible material for the construction of stomatological and orthopedic implants) with atoms of fullerene C_60_, which can act as very efficient radical scavengers. However, fullerenes and their derivatives are able to generate harmful reactive oxygen species and to have cytotoxic effects. In order to stabilize C_60_ molecules and to prevent their possible cytotoxic effects, deposition in the compact form of Ti/C_60_ composites (with various Ti concentrations) was chosen. The reactivity of C_60_/Ti composites may change in time due to the physicochemical changes of molecules in an air atmosphere. In this study, we therefore tested the dependence between the age of C_60_/Ti films (from one week to one year) and the adhesion, morphology, proliferation, viability, metabolic activity and potential DNA damage to human osteosarcoma cells (lines MG-63 and U-2 OS). After 7 days of cultivation, we did not observe any negative influence of fresh or aged C_60_/Ti layers on cell behavior, including the DNA damage response. The presence of Ti atoms resulted in improved properties of the C_60_ layers, which became more suitable for cell cultivation.

## Introduction

Fullerenes are spheroidal hollow cage-like carbon nanoparticles with diverse biological activities. Due to their unique physicochemical properties, e.g. the ability to withstand high temperatures and pressures, and also the high reactivity of these nanoparticles, fullerenes are expected to have great potential in a wide range of fields including medicine. The high reactivity of these molecules has been explained by bending of sp^2^-hybridized carbon atoms, which produces angle strain, and by the presence of double bonds, which can react with radical species. Fullerenes C_60_ and their derivatives are therefore considered to be the world’s most efficient radical scavengers with strong antioxidant properties (for a review, see [1, 2]). For example, fullerene C_60_ and its derivative fullerol has been reported to antagonize the oxidative stress generated by dexamethasone therapy, and thus to prevent osteonecrosis [3, 4]. By quenching oxygen radicals, fullerenes C_60_ also inhibit the differentiation of osteoclasts and the production of matrix metalloproteases, and can thus inhibit the destruction of bone and cartilage tissue in arthritis [5, 6]. Complexes of fullerenes with polyvinylpyrrolidone (with fullerene C_60_ as the major component) displayed photoprotective effects on keratinocytes against ultraviolet B irradiation [7].

However, fullerenes are able not only to quench, but also to generate dangerous reactive oxygen species (ROS). Numerous studies have described fullerenes as a cytotoxic and genotoxic agent, causing oxidative DNA damage [8, 9], inhibition of detoxificatory and antioxidant enzymes [10], polyploidy [11], premature cell senescence [12], apoptosis [13] and inflammation [14]. The biological response to fullerenes is profoundly influenced by their physical and chemical properties, such as water solubility [15]; for a review, see [16], functionalization with various chemical groups [17], electronic behavior, degree of agglomeration [13], and also concentration [14]. For example, increased water solubility was associated with decreased cytotoxicity of C_60_. On the other hand, certain solvents can enhance fullerene toxicity (for a review, see [16]). The carboxylate derivatization of fullerenes was the determining factor in their ability to induce apoptosis in human monocytic THP1 cells [13]. At lower concentrations (less than 0.04mg/ml), fullerene-based amino acid nanoparticles 0.04 mg/mL initiated less cytokine activity and maintained the viability of human keratinocytes, while at higher concentrations (0.04 to 0.4 mg/ml) these nanoparticles were cytotoxic and pro-inflammatory [14].

In order to prevent possible cytotoxic effects of fullerenes, deposition of these molecules in the form of compact and stable layers, well-adhering to the underlying substrate, was chosen. We supposed that the fullerene films could be strengthened by introducing a biocompatible metallic component into the films. Fullerene C_60_-gold nanoparticle films, self-assembled on silanized glass coverslips, showed good chemical and ultrasonic stability, as revealed by their immersion in 0.1 M HCl and by their exposure to ultrasonic irradiated surrounding [18]. The introduction of a suitable metallic component was expected to stabilize the fullerene films in terms of reducing the release of free C_60_, their penetration into cells, and thus to eliminate the potential negative effects of fullerenes. Titanium was chosen as this metallic component, due its biocompatibility, which has been proven in its numerous and long-lasting experimental and clinical applications. Titanium is a metal that has been widely used for constructing stomatological implants and, in the form of alloys, such as Ti-6Al-4V or newly developed beta-titanium alloys, also for orthopedic implants, such as load-bearing joint replacements [19–22]; for a review, see [23]. Titanium was also tested with positive results in our earlier studies as a potential component of carbon-based coatings of bone implants, namely amorphous carbon with titanium [24] or hydrocarbon plasma polymers enriched with Ti [25]. Specifically, the presence of Ti in these coatings enhanced the adhesion, spreading, growth and production of osteocalcin in human osteoblast-like MG-63 cells. The presence of Ti in diamond-like carbon (DLC) coatings also increased their bioactivity compared to pure DLC. This was manifested by precipitation of compounds containing calcium and phosphorus, i.e., basic components of the bone apatite, and by increased colonization of Ti-doped DLC with human osteoblast-like MG 63 cells [26]. At the same time, the addition of Ti into DLC coatings improved their mechanical properties, namely by increasing their adhesion to the underlying substrates [27], by decreased their residual stress and friction coefficient, and by modulating their hardness to appropriate values [28, 29].

The construction of C_60_/Ti composites in this study was also inspired by our earlier studies and by studies by other authors, in which C_60_ was combined with transitional metals, namely Ni, Fe, Nb, Pt and Pd [30–34]. These composites showed interesting structural, electrotransport, electrochemical and photoelectric properties, and are applicable in electronics or photovoltaics [35]; for a review, see [33]. However, with the exception of Nb, which is considered as biocompatible, all metals mentioned here are known to be cytotoxic. To the best of our knowledge, C_60_/Ti composite films, with the exception of our earlier studies [36, 37], have not yet been constructed and investigated for biomedical purposes by other authors. In our earlier studies, only the adhesion and growth of MG-63 cells, measured by changes in their number in three time intervals, were investigated on C_60_/Ti composite films, together with regional selectivity of cell colonization, if these films were constructed as micropatterned, i.e. containing grooves and prominences [36, 37]. The novelty of present study lies in the deeper investigation of the cell behavior on C_60_/Ti films, including not only their adhesion and growth, but also their viability, mitochondrial activity, and potential DNA damage.

Another important factor investigated in our study is the influence of the age of C_60_/Ti composites on these parameters, as well as on the regional selectivity of the cell colonization on films with a micropatterned morphology. In our earlier study performed on pure fullerene C_60_ films, fresh fullerene films lowered the cell number, viability, growth and metabolic activity, and these parameters improved markedly with aging of the C_60_ films [38]. Moreover, micropatterned fresh fullerene films promoted regionally-selective cell colonization in grooves among the prominences, which almost disappeared on aged fullerene films. These results were attributed to changes in the fullerene films during aging, e.g. fragmentation, oxidation, polymerization and graphitization of fullerenes in an air atmosphere, and thus loss of their reactivity [38].

Last but not least, the C_60_/Ti fullerene films in the present study were deposited with three different concentrations of Ti, ranging from ~ 25% (i.e., 25 Ti atoms and 75 C_60_ molecules) to ~ 70%, in order to investigate potential differences in their stability and in the cell behavior on these surfaces. On DLC films doped with three concentration levels of Ti (up to 23 at. %), the number of MG-63 cells increased with the increasing Ti concentration. They were highest on DLC with a medium and highest content of Ti [26].

## Material and Methods

### Material deposition

**Fig 1:**
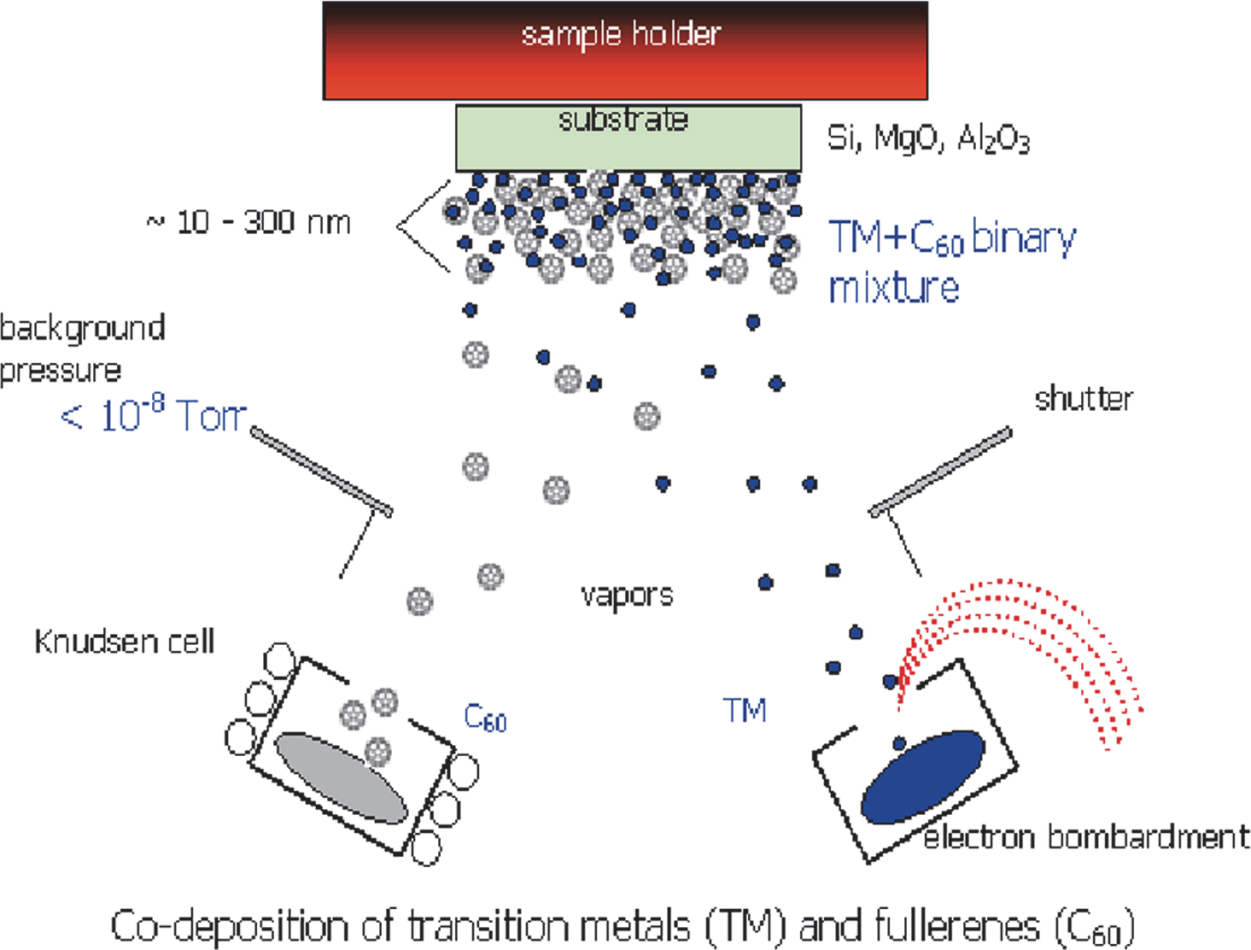
Scheme of the preparation of hybrid fullerene C_60_/metal composites. Deposition rates: DR(M) = DR(C_60_) ~ 1 nm/min. Temperatures during deposition: RT.

The C_60_/Ti composite films were prepared by co-deposition of C_60_ and Ti onto microscopic glass coverslips (Menzel-Gläser, Germany, diameter 12 mm) in the Molecular Beam Epitaxy (MBE) chamber using the Knudsen cell and an e—gun for vaporization of C_60_ and Ti, respectively (**Fig 1**), under certain deposition kinetics: background pressure during deposition ~ 5 × 10^−7^ Torr; deposition rate ~ 1 nm/min; temperature of the substrates during deposition ~ room temperature (RT). Three C_60_/Ti systems, with different phase ratios, were fabricated, i.e., with a low concentration (25%, i.e., 25 Ti atoms and 75 C_60_ molecules), medium concentration (45%) and high concentration (70%) of Ti atoms in the composite.

The composites were synthesized with a micropatterned morphology by deposition through a contact mask (a metallic mesh) producing rectangular C_60_/Ti prominences with an average size of 128 μm per 98 μm (12,500 μm^2^) and with 50 μm spacing. However, as revealed by Raman spectroscopy and AFM, these spaces (grooves) also contained a very thin continuous film of C_60_/Ti composites.

The samples were stored for 1–2 weeks (fresh samples) or for 1 year (aged samples) in an air atmosphere at room temperature in a dark and dry place, and were then evaluated.

### Raman spectroscopy

A Renishaw 2000 imaging microscope (using a 514 nm Ar laser) was applied for an analysis of the C_60_/Ti films. The measurements were performed using low laser power, i.e., (< 1 mW) in order to avoid fragmentation of the C_60_ molecules. The spectra were measured on the top of the C_60_/Ti prominences, using multi-peak Gaussian analysis of the H_g_(7), A_g_(2) and H_g_(8) vibration peaks. Area peak ratios A_g_(2)/H_g_(7) and A_g_(2)/H_g_(8) were evaluated.

### Atomic force microscopy (AFM)

The surface morphologies of the C_60_/Ti layers were analyzed by atomic force microscopy (AFM microscope NTEGRA, NT-MDT) using a static (contact) mode. The scanning area was selected as 5 x 5 μm^2^.

### Stability of C_60_/Ti coating (potential water dissolution)

All examined C_60_/Ti coatings with a low, medium and high content of Ti were incubated in 1 ml of deionized water at 37°C in a humidified air atmosphere containing 5% of CO_2_ for 24 hours (mimicking the rinsing phase prior to use for all biological experiments; described below in Cells and culture conditions). After 24 hours, the water was transferred from the C_60_/Ti samples to glass Petri dishes (diameter 2 cm), and fresh deionized water was added to the same C_60_/Ti samples for further 48 hour-long incubation (mimicking the incubation phase with cells in biological experiments). The water solutions were slowly dried on glass Petri dishes for 2 days. When all water was evaporated, the thin films that had formed on the bottom of the Petri dishes were analyzed by Raman spectroscopy.

### Measurement of wettability

The surface wettability of the C_60_/Ti composites was estimated from the contact angle measured by a material-water droplet system using a reflection goniometer (SEE System, Masaryk University, Brno, Czech Republic). The data was presented as mean ± standard error of the mean (S.E.M.) obtained from 10 measurements.

### Cells and culture conditions

Since the samples were prepared under aseptic conditions (assured by the high temperature), sterilization was not performed in order to avoid potential damage to the fullerene molecules by irradiation, heating or chemicals. However, to prevent the potential release of newly deposited C_60_/Ti molecules into the culture medium, all samples (i.e., glass coverslips coated with C_60_/Ti films with various Ti concentrations) were incubated in deionized water at 37°C in a humidified air atmosphere containing 5% of CO_2_ for 24 hours prior to each biological experiment. The samples were then repeatedly rinsed in phosphate-buffered saline (PBS; Sigma, Missouri, U.S.A.). For studies on cell adhesion, spreading, growth, morphology, viability and metabolic activity, the samples were seeded with human osteoblast-like MG-63 cells (European Collection of Cell Cultures, UK) in an initial density of 5 370 cells/cm^2^ (10 000 cells per well). For studies on DNA damage, human osteoblast-like U-2 OS cells (ATCC-LGC, No. HTB-96) were used in densities ranging from 4 300 cells/cm^2^ (8 000 cells per well) to 16 100 cells/cm^2^ (30 000 cells per well). Both cell lines were cultured for 1, 3 or 7 days in 1 mL of Dulbecco's Modified Eagle's Medium (Sigma, Missouri, U.S.A., Cat. No. D5648) supplemented with 10% fetal bovine serum (Sebak GmbH, Germany) and gentamicin (40 μg/mL; LEK, Slovenia) at 37°C in a humidified air atmosphere containing 5% of CO_2_. Uncoated microscopic glass coverslips (Menzel-Gläser, Germany; diameter 12 mm) were used as a reference material. For each experimental group and time interval, 3 samples were analyzed, and the experiment was repeated three times.

### Evaluation of cell morphology, initial adhesion and proliferation

The MG-63 cells were cultured for 7 days (seeding density 5 370 cells/cm^2^; 10 000 cells per well). An evaluation of the cell morphology was performed on days 1, 3 and 7 after seeding, using an IX-71 microscope equipped with a DP-71 digital camera (Olympus, Japan). Immediately after that, each sample was transferred to fresh polystyrene 24-well tissue culture plates and rinsed with PBS. The cells were detached by a trypsin-EDTA solution (Sigma, Missouri, U.S.A., Cat. No T4174) and were counted using a Bürker haemocytometer (days 1 and 3) or using a Vi-Cell XR analyzer on day 7 (Beckman Coulter, California, U.S.A.). The cell numbers were expressed as cell population densities/cm^2^ and were also used for calculating the cell population doubling time according to the following formula:

$$DT=\log\;2\frac{t-t_{0}}{\log N_{t}-\log N_{t}{}_{{}_{0}}}$$

where *t*_0_ and *t* represent earlier and later time intervals after seeding, respectively, and *N*_t0_ and *N*_t_ are the numbers of cells at these intervals.

In order to confirm the validity of the results, the experiments were repeated and data from separate experiments was analyzed. For each experimental group and time interval, three parallel samples were evaluated, and the experiment was repeated three times.

### Evaluation of cell metabolic activity

The commercial Cell Proliferation Kit II XTT (Roche, Switzerland, Cat.No.11 465 015 001) was used to investigate the potential cytotoxicity of the C_60_/Ti films. This is a set of colorimetric assays based on cleavage of the yellow tetrazolium salt XTT (2,3-bis(2-methoxy-4-nitro-5-sulphophenyl)-2H-tetrazolium-5-carboxanilide) to a soluble orange formazane derivate by mitochondrial enzymes from metabolically active cells (an indirect measure of the cell proliferation activity). The formazane dye is directly quantified by a spectrophotometer. After 3 and 7 days of cultivation, all samples were transferred to new polystyrene 24-well tissue culture plates and were rinsed with PBS. A 1 mL solution of XTT and Dulbecco's Modified Eagle's Medium without Phenol Red (Gibco, Cat. No 11053–028), supplemented with 10% fetal bovine serum (Sebak GmbH, Germany) and gentamicin (40 μg/mL; LEK, Slovenia) in the ratio of 1 volume part of XTT to 2 volume parts of DMEM, was added to each sample (according to the manufacturer’s protocol). After 4–6 hours of incubation at 37°C in a humidified air atmosphere containing 5% of CO_2_, the absorbance of the resulting solution was measured at wavelength 470 nm against the reference value of 650 nm.

Solutions from C_60_/Ti-coated samples and also from uncoated microscopic glass coverslips without seeded cells were used as blank samples. In order to confirm the validity of the results, the experiment was repeated three times, and the data from separate experiments was analyzed. For each experimental group and time interval within one experiment, three parallel samples were used and the solution from each well was divided into 8 parallel wells.

### Evaluation of membrane damage and cell viability

On day 7 after seeding, cell viability and membrane damage to cells were detected by trypan blue staining performed during cell counting in the Vi-CELL XR analyzer (Beckman Coulter, California, U.S.A.). Data from three separate experiments was analyzed. For each experimental group, 50 images from three parallel samples were evaluated within one experiment.

### Evaluation of the DNA damage response

In order to investigate potential DNA damage to the cells, osteosarcoma cell line U-2 OS was used instead of MG-63, which is p53 deficient. After 3 and 7 days of cultivation, the DNA damage response was evaluated by immunofluorescence staining analyzed by fluorescence microscopy and flow cytometry.

The samples for microscopy were rinsed with PBS and fixed with 4% paraformaldehyde (PFA; Sigma, Missouri, U.S.A.) for 20 minutes at room temperature. Subsequently, the cells were permeabilized with 0.1% Triton X-100 in PBS (Sigma, Missouri, U.S.A.) for 20 minutes at room temperature. This solution also contained 1% bovine serum albumin for blocking non-specific binding sites for antibodies. The samples were incubated with primary antibodies anti-53BP1 (0.2 μg/mL; Santa Cruz Biotech, California, U.S.A.; clone H-300) and anti-H2A.X-Phosphorylated Ser139 (0.4 μg/mL; Millipore, Massachusetts, U.S.A.; clone JBW301) for 1 hour, followed by secondary antibodies coupled to Alexa Fluor 488 and 546 (4 μg/mL; Invitrogen, Molecular Probes, Oregon, U.S.A.) for 1 hour. The cells were then mounted with a microscopic glass coverslip using a Gel/Mount permanent fluorescence-preserving aqueous mounting medium (Biomeda Corporation, California, U.S.A.) and were evaluated under the IX-71 epifluorescence microscope (Olympus, Japan) equipped with a DP-71 digital camera (Olympus, Japan).

The samples analyzed by flow cytometry were prepared using the same protocol as those for microscopy, except that all steps were performed in a suspension, not on microscopic glass coverslips. Alexa Fluor 488 anti-H2A.X-Phosphorylated (Ser139) antibody (5 μg/1 million cells; BioLegend, California, U.S.A.; clone 2F3) was used for flow cytometry. After 1 hour of incubation with antibody, the cells were rinsed and resuspended in PBS. The samples were analyzed using an Accuri C6 Flow Cytometer (BD Biosciences, New Jersey, U.S.A.).

U-2 OS treated with neocarzinostatin (NCS; 700 ng/mL; Sigma, Missouri, U.S.A.) for 1 hour were used as a positive control to markers of a DNA damage response. The cells were fixed 3 hours after treatment with NCS. Immunofluorescence staining and also flow cytometry analysis were repeated in order to confirm the results.

### Statistical analysis

The data was presented as mean ± S.E.M. (Standard Error of the Mean) or median with interquartile range (IQR) obtained from three separate experiments. Within each experiment, three samples for each experimental group and time interval were evaluated. A comparison between all groups was analyzed by two-way ANOVA, Student-Newman-Keuls Method, to evaluate two factors: the composition of the C_60_/Ti layers (Ti content—25%, 45%, 70%) and their age (1 week or 1 year). *P*-values less than 0.05 were considered statistically significant.

## Results and Discussion

### Raman spectroscopy

**Fig 2:**
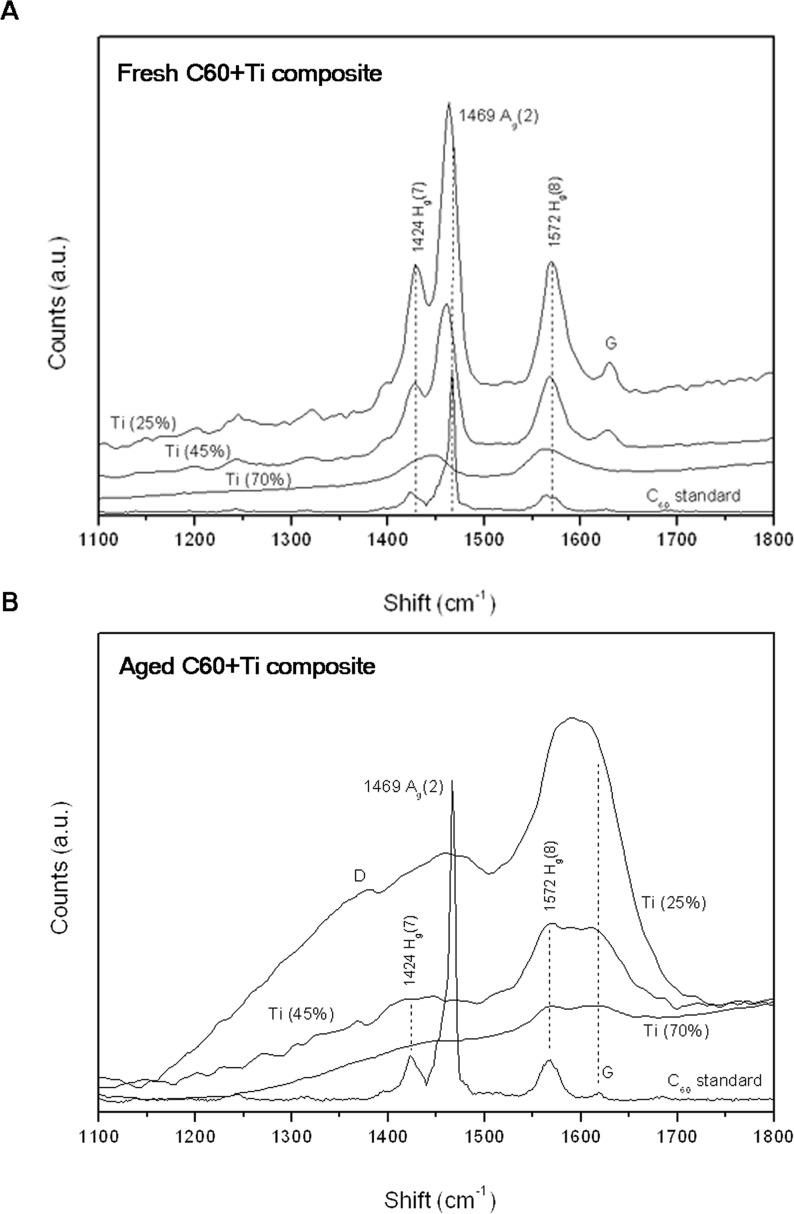
Raman spectra (between 1100–1800 cm^-1^) of the fresh *(A)* and aged *(B)* C_60_/Ti composites with various Ti concentrations (low: 25%, medium: 45%, high: 70%). For comparison, a spectrum from the C_60_ standard is shown.

A study by Raman spectroscopy measured on the top of the prominences revealed a change in both the fresh and aged fullerene films, in comparison with the C_60_ standard. **Fig 2A** depicts the Raman spectra (only relevant details between 1100–1800 cm^-1^) measured on the fresh (i.e., 1-week-old) C_60_/Ti composites deposited on glass coverslips at RT with a low (25%), medium (45%) and high (70%) concentration of Ti. For comparison, a Raman spectrum from the C_60_ standard (a film of C_60_ deposited on glass coverslips) is also shown. The main Raman vibration modes for fullerenes: A_g_(2), H_g_(7) and H_g_(8) were inspected. For all fresh layers, a change in the spectra revealed that the intensity of the most important A_g_(2) peak (pentagonal pinch mode, characteristic for fullerenes) dropped dramatically down (compared to the neighboring H_g_(7) and H_g_(8) vibration modes). Moreover, the A_g_(2) peak exhibits a significant red shift towards position 1450 cm^-1^. The H_g_(7) and H_g_(8) modes remained on the same positions, but the area ratios H_g_(7)/A_g_(2) and H_g_(8)/A_g_(2) altered in comparison with the C_60_ standard. These changes are ascribed to alterations in the chemical bonding of C_60_, such as polymerization (interaction of fullerene molecules into a polymerized network) and oxidation (chemical bonding of oxygen with fullerene molecules) [39]. It is known that A_g_(2) is the most sensitive vibration mode—by analyzing this mode one can get information about the C_60_ structural and bonding change (high sensitivity of Ti/C_60_ towards oxidation is described e.g. in [30]). All these changes are more obvious with increasing Ti content. In addition, new vibration modes G appeared in layers with a low and medium Ti content, indicating the formation of graphitic flakes.

An examination of aged (i.e., 1-year-old) C_60_/Ti composites revealed dramatic difference in comparison with the fresh samples. The most important A_g_(2) mode is suppressed for all Ti concentrations, and H_g_(8) and G (formation of graphitic flakes and fragmentation of fullerenes) became the most prominent peaks. Another important difference of aged layers is the formation of the D band in films with low and medium Ti content, indicating disordered nanocarbon with sp^3^ bonding (**Fig 2B**).

Degradation and oxidation of C_60_ films during aging was also proven by X-ray Photoelectron Spectroscopy (XPS) in our earlier study [38]. Alterations and fragmentation of fullerenes are enhanced in hybrid systems (transition metal/C_60_) because of the strong catalytic properties of transition metals (including Ti) during co-deposition, which may cause the fullerene decay. The effect of aging was therefore least obvious in the samples with a high Ti concentration. Based on reports from a similar system (with the combination of immiscible phases, i.e., C_60_/Ni), the transition metal–fullerene hybrid composites, deposited at RT, are structurally stressed and exhibit a tendency toward phase separation [31, 32]. The final structure depends on the ratio of the building blocks (Ti and C_60_), the thickness of the film and the temperature of preparation. Obviously, the different structure with different chemical bonds and surface morphology can have a different effect on the adhesion and growth of cells in biological systems.

### Atomic force microscopy (AFM*)*

**Fig 3:**
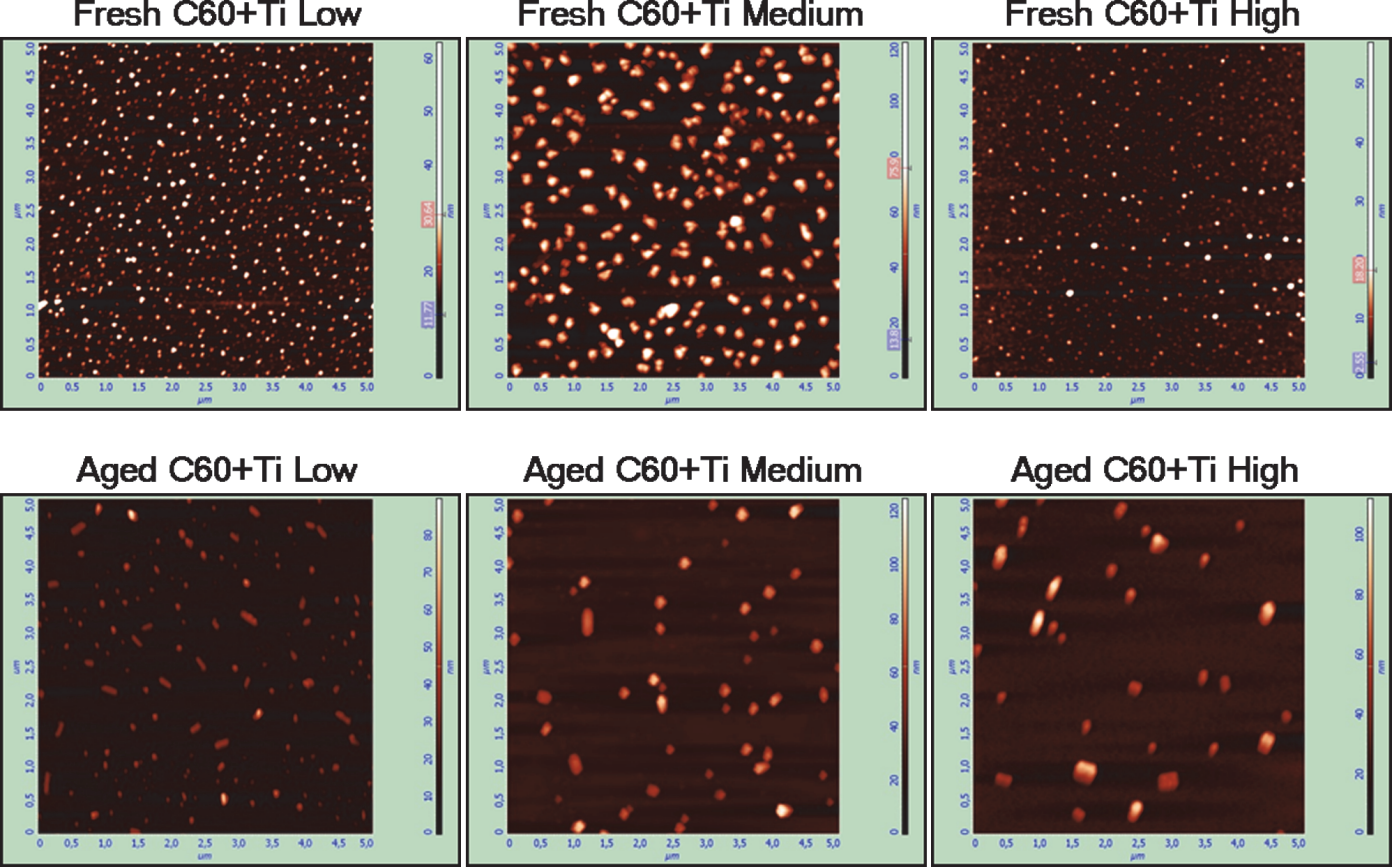
AFM images of the surface morphology on the prominences of the fresh and aged C_60_/Ti composites with various Ti concentrations (low: 25%, medium: 45%, high: 70%).

The morphology of the C_60_/Ti films was analyzed by AFM (**Fig 3**) in the 5 x 5 μm^2^ scanning areas. The thickness of the films varied from 10 nm (in grooves among the prominences) to about 300 nm (on the tops of the prominences). The ratio between the thicknesses of the C_60_/Ti films at the prominences and in the grooves changed in the course of time, i.e., the ratio became lower in the aged composites (**Table 1**). This could be explained by a decrease in the height of the prominences after diffusion of the fullerenes, which was also observed in our earlier studies performed on pure micropatterned C_60_ films [38, 40].

**Table 1:** 

****Parameter****	****Fresh C****_60_ ****/Ti films****	****Aged C****_60_ ****/Ti films****
TP_25_ / TG_25_	~ 8.3	~ 3.5
S_25_ [nm]	50–100	50–200
D_25_ [particles / 25 μm^2^]	~ 650	~ 150
TP_45_ / TG_45_	~ 10	~ 5.3
S_45_ [nm]	100–200	50–200
D_45_ [particles / 25 μm^2^]	~ 250	50
TP_70_ / TG_70_	~ 11.6	~ 4
S_70_ [nm]	25–75	50–150
D_70_ [particles / 25 μm^2^]	~ 250	30

Main characteristics of the surface morphology of the prominences for fresh and aged (1-year-old) C_60_/Ti thin films with various Ti concentrations (low: 25%, medium: 45%, high: 70%). TP/TG—ratio between the thickness of the prominences and the thickness of the grooves, S—particle size, D—particle area density. Scanning areas: 5 x 5 μm^2^.

In addition, the AFM images from the top of the prominences of all deposited systems with low (25%), medium (45%) and high (70%) atomic concentrations of Ti exhibited an interesting feature—the formation of particles with a different size (*S*) and area density (*D*), see **Table 1**. These particles, grown on the surface of the samples, were inspected by Raman spectroscopy and it was confirmed that they are large fullerene clusters with a low concentration of the Ti phase (causing only a mild disruption of the A_g_(2) pinch mode). This interesting effect was already observed for the C_60_/Ni hybrid system that was also prepared at RT [34]. The C_60_/Ni composite was grown as a stressed, supersaturated mixture of two immiscible phases showing a strong proclivity to phase separation and particle network formation. After a year, about 200 particles per mm^-2^ several micrometers in size were formed. Interestingly, in the case of the C_60_/Ti composites, all the observed particles had become smaller in size, the largest being only about 200 nm. The reason might be a limited reservoir (from a single prominence) for the diffusing fullerene molecules (building blocks for the grown particles), or different stress intensity (in comparison to the C_60_/Ni system). On the other hand, the size of the C_60_/Ti particles was larger than the typical granule size observed on pure C_60_ layers (~50 nm) [38]. In both (fresh and aged) cases, however, the morphology of the prominences changed, and this new nonostructural surface may be expected also to affect the biocompatibility of the hybrid system.

In addition, a different density of these clusters was observed on fresh and aged C_60_/Ti layers. The number of C_60_/Ti particles was higher on the fresh layers than on the aged films, but the size of the particles was slightly higher in the aged films (**Fig 3, Table 1**). This could be explained by fragmentation and diffusion of the fullerene molecules, and also by their polymerization and other changes in the C_60_/Ti systems during the aging period.

### Stability of C_60_/Ti coating in a water environment

**Fig 4:**
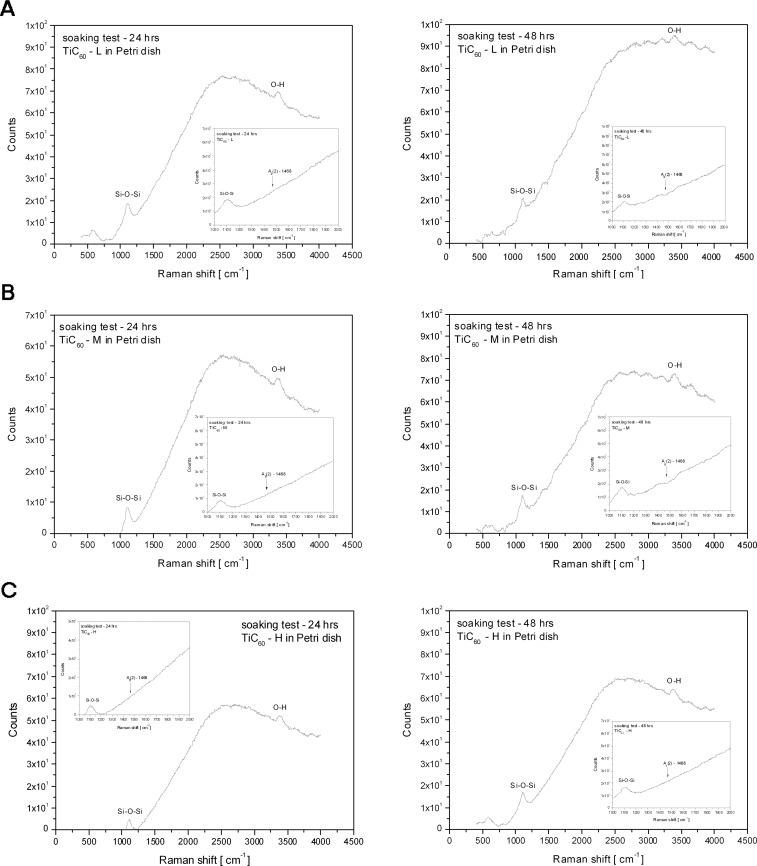
Raman spectrum of thin films formed in Petri dishes by evaporating water solutions after incubation of C_60_/Ti composites with a low *(A)*, medium *(B)* and high *(C)* content of Ti for 24 hours and then for another 48 hours. No A_g_(2) vibration mode (i.e., no presence of C_60_) was confirmed.

The stability of fresh C_60_/Ti layers with a low, medium and high content of Ti was evaluated by dissolution in deionized water, and was analyzed by Raman spectroscopy. In the original (as deposited) films, the Raman measurements point to the dominant A_g_(2) breathing mode, confirming the presence of fullerenes (**Fig 2**; described above). However, the examination of the dried water in the Petri dishes, taken from the C_60_/Ti films, did not prove any presence of fullerenes or other carbon allotropes. The Raman spectra showed only a broad luminescence distribution with Si-O-Si and O-H peaks from glass (Petri dish) and H_2_O (**Fig 4**). Thus, no dissolution of C_60_ molecules was observed, and all tested C_60_/Ti films deposited on the glass coverslips were mechanically stable in the water.

### Hydrophobicity of C_60_/Ti layers

**Fig 5:**
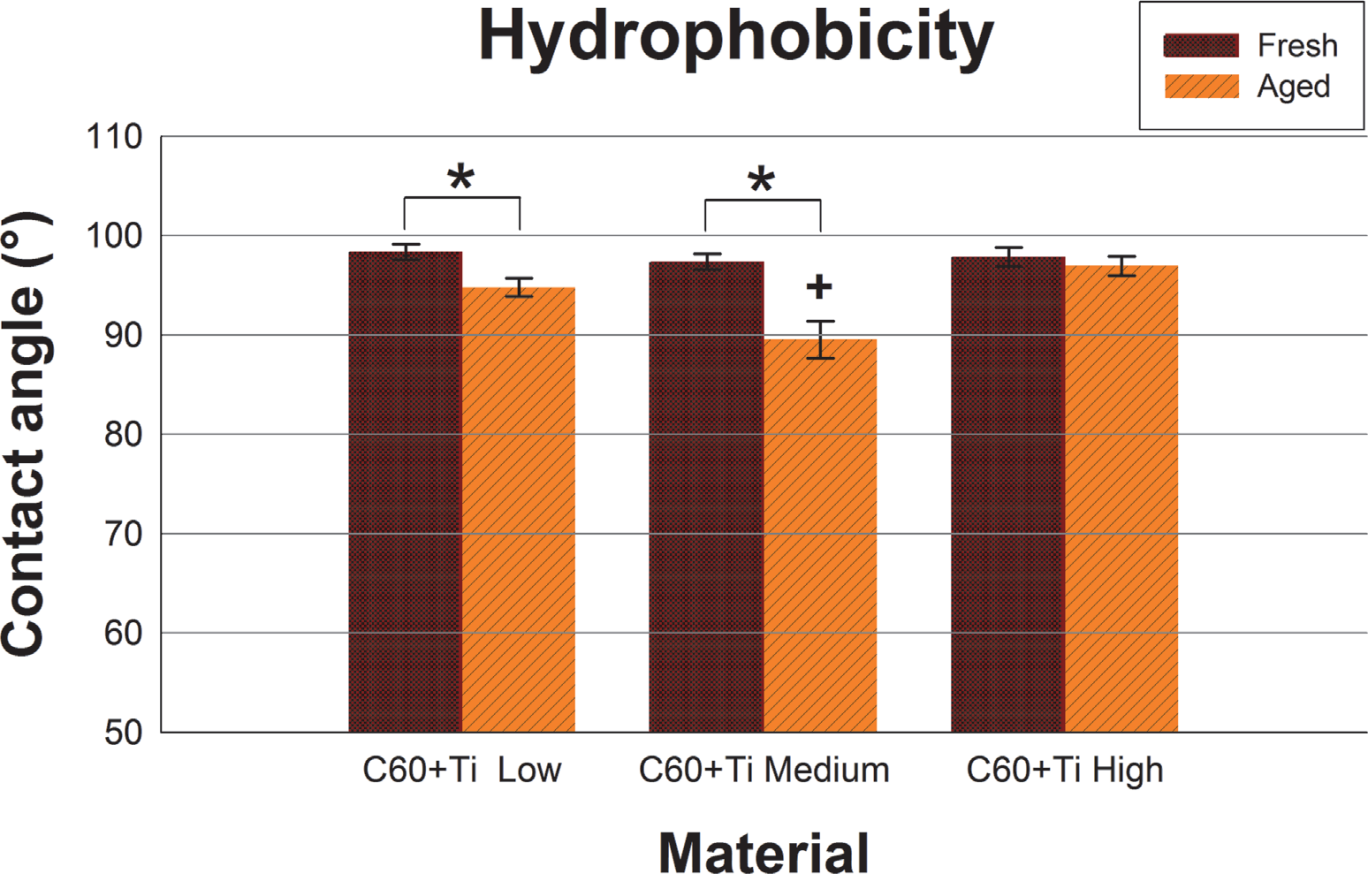
Static water drop contact angle of fresh and aged C_60_/Ti composites with various Ti concentrations (low: 25%, medium: 45%, high: 70%). * significant difference between fresh and aged layers; p ≤ 0.05.

Both fresh and aged layers of all C_60_/Ti composites with various Ti concentrations were at a relatively high hydrophobic level ranging from 89.6° to 98.4°. A significant decrease in the water contact angle was observed during the aging of C_60_/Ti films with low and medium Ti content (**Fig 5, S1 Table, S1 Dataset**).

This could be explained by spontaneous physicochemical changes (such as fragmentation, polymerization, oxidation and graphitization) in an air atmosphere (**Fig 2**; described above). This decline was not observed for aged C_60_/Ti layers with a high Ti content (**Fig 5, S1 Table, S1 Dataset**), which is in correlation with the results obtained from Raman spectroscopy, where the spectra of the fresh and aged samples were very similar, and therefore fewer physicochemical changes occurred during their aging (**Fig 2**; described above). The presence of oxygen and the formation of oxygen-containing chemical functional groups are known to increase the surface wettability of various materials, e.g. synthetic polymers, metals or carbon-based materials (for a review see [1, 2]). In our earlier studies, oxidation and also fragmentation, polymerization and graphitization of fullerenes were observed on fullerene films exposed to 70% cold ethanol used for material sterilization.

### Initial adhesion, proliferation and morphology of cells on fullerene C_60_ /Ti layers

**Fig 6:**
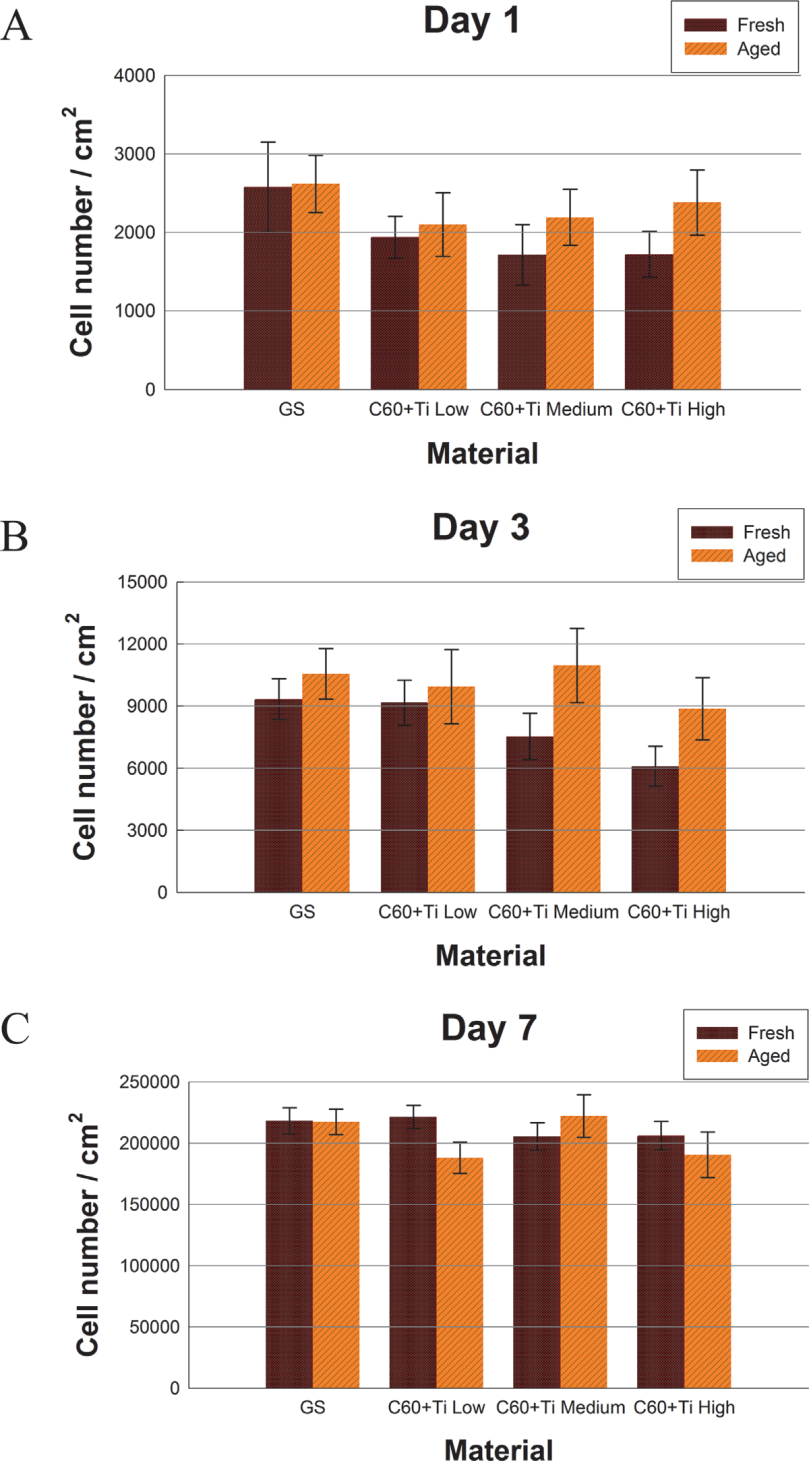
Numbers of human osteoblast-like MG-63 cells on fresh or aged C_60_/Ti composites with various Ti concentrations (low: 25%, medium: 45%, high: 70%) on day 1 *(A)*, 3 *(B)* and 7 *(C)* after seeding. GS: microscopic glass coverslips, a reference material. No significant differences among the experimental groups were found.

The number of initially adhered human osteoblast-like cells MG-63 cells on day 1 after seeding on fresh (i.e., one-week-old) C_60_/Ti composites with various Ti additions was slightly lower (the decrease correlated positively with the increase in Ti concentration) in comparison with the reference microscopic glass coverslips (**Fig 6A, S2A Table, S1 Dataset**); however, these reductions were not proven to be statistically significant. Similar results for cell numbers were obtained on day 3 after seeding (**Fig 6B, S2B Table, S1 Dataset**). The calculation of the cell population doubling time also did not reveal any significant decrease in proliferation of cells cultured on fresh C_60_/Ti films. The doubling times of the cells cultured on all fresh samples were comparable with the reference material (**S1 and S2 Figs, S3 Table, S1 Dataset**).

On the aged C_60_/Ti films, the initial adhesions as well as the cell numbers on all tested samples were almost the same in all culture intervals (**Fig 6, S2 Table, S1 Dataset**). The growth dynamics of cells cultured on aged composites of all Ti concentrations was also similar to that on the reference material (the doubling times are shown in **S1 and S2 Figs, S3 Table, S1 Dataset**).

In a previous study performed on pure C_60_ layers, lower numbers and slower proliferation of MG 63 cells were found on the fresh films in comparison with the control glass coverslips or with aged C_60_ films [38]. However, on aged C_60_ films, these differences diminished considerably or almost disappeared. This result was explained by changes in C_60_ molecules during their ageing, such as fragmentation, polymerization and oxidation, which decreased the reactivity of fullerenes. Interestingly, the examination of C_60_/Ti composites in this study showed no significant differences in cell adhesion and growth between the fresh and aged films. Moreover, from this point of view, both fresh and aged layers were comparable to the reference glass coverslips. A possible explanation for this improvement of the fresh C_60_ layers for cell cultivation by co-deposition with Ti could be that fragmentation, polymerization and oxidation of C_60_ occurred during deposition of the composite films by the interaction of C_60_ molecules with Ti atoms, and not only due to the ageing of the C_60_/Ti films. In other words, the C_60_/Ti composites exhibited similar biocompatibility as the mix of amorphous carbon and titanium. Similarly, amorphous carbon in the form of films or electrospun nanofibrous scaffolds has been shown to provide good support for the adhesion and proliferation of mouse neuroblastoma N2a cells and rat Schwann RT4-D6P2T cells [41].

**Fig 7:**
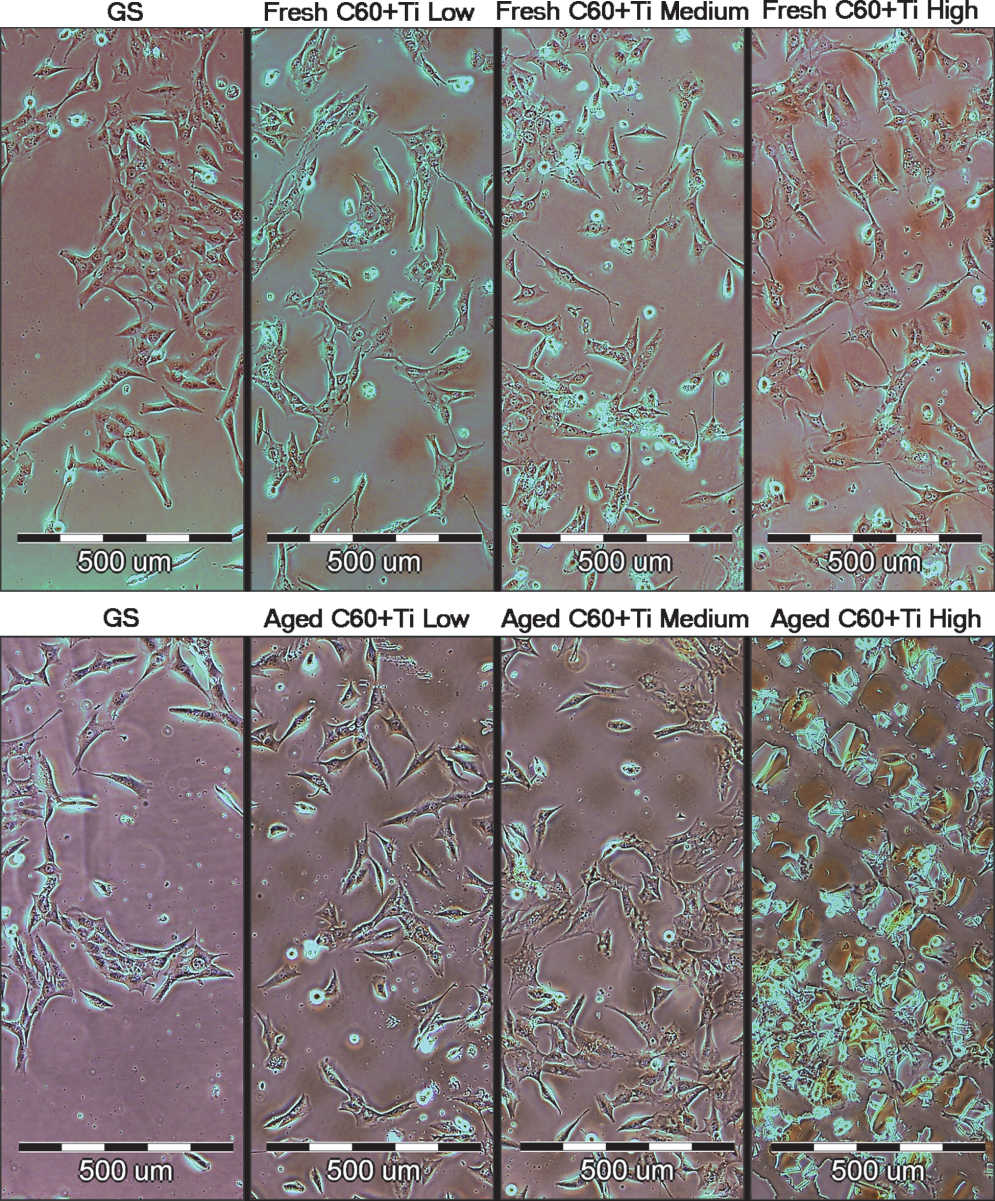
Morphology of human osteoblast-like MG-63 cells on day 3 after seeding on fresh and aged C_60_/Ti composites with various concentrations of Ti (low: 25%, medium: 45%, high: 70%). GS: microscopic glass coverslips, reference material.

The cell morphology was similar on both fresh and aged C_60_/Ti composites of all Ti concentrations. The cells were generally well-spread, polygonal or spindle-shaped. No cytotoxic morphological changes, such as enlarged cells or cytosolic vacuole formation, were observed on fresh or on aged C_60_/Ti films with various Ti concentrations (**Fig 7**).

Preferential growth in grooves among the prominences was also apparent, particularly on the fresh composites (**Fig 7**). Similar cell behavior was also observed in our earlier studies performed on micropatterned pure C_60_ as well as hybrid C_60_/Ti films [36–38, 40]. Nevertheless, on micropatterned pure C_60_ films, the preferential cell colonization in grooves was much more apparent on the fresh films than on the aged films. This was explained by the diffusion of C_60_ molecules from the prominences towards the grooves and thus lowering of the prominences during aging [38]. On the composite C_60_/Ti films in the present study, prominences and preferential cell colonization in grooves were still apparent on the aged films, particularly those with the highest Ti concentration. This could be attributed to increased stability of the prominences due to the presence of Ti atoms.

### Metabolic activity and viability of cells on fullerene C_60_ /Ti layers

**Fig 8:**
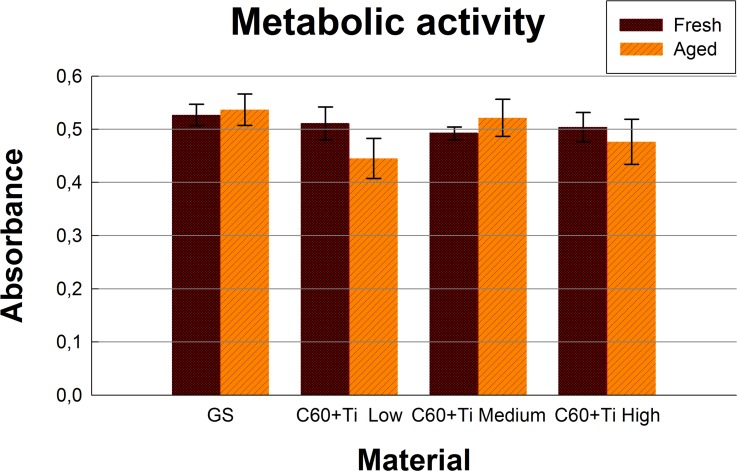
Metabolic activity measured by the XTT test per culture of human osteoblast-like MG-63 cells on day 7 after seeding on fresh and aged C_60_/Ti composites with various Ti concentrations (low: 25%, medium: 45%, high: 70%). GS: microscopic glass coverslips, a reference material. No significant differences among the experimental groups were found.

In order to investigate the potential cytotoxicity of fresh and aged C_60_/Ti composites with various Ti additions, an XTT cell proliferation assay was performed. Proportionally to the cell numbers, MG-63 cultivated for 7 days on both fresh and aged C_60_/Ti layers showed comparable metabolic activity (i.e., activity of mitochondrial enzymes) with cells grown on control glass coverslips (**Fig 8, S4 Table, S1 Dataset**). No significant differences in metabolic activity were found among the various Ti concentrations or the ages of the C_60_/Ti composites.

**Fig 9:**
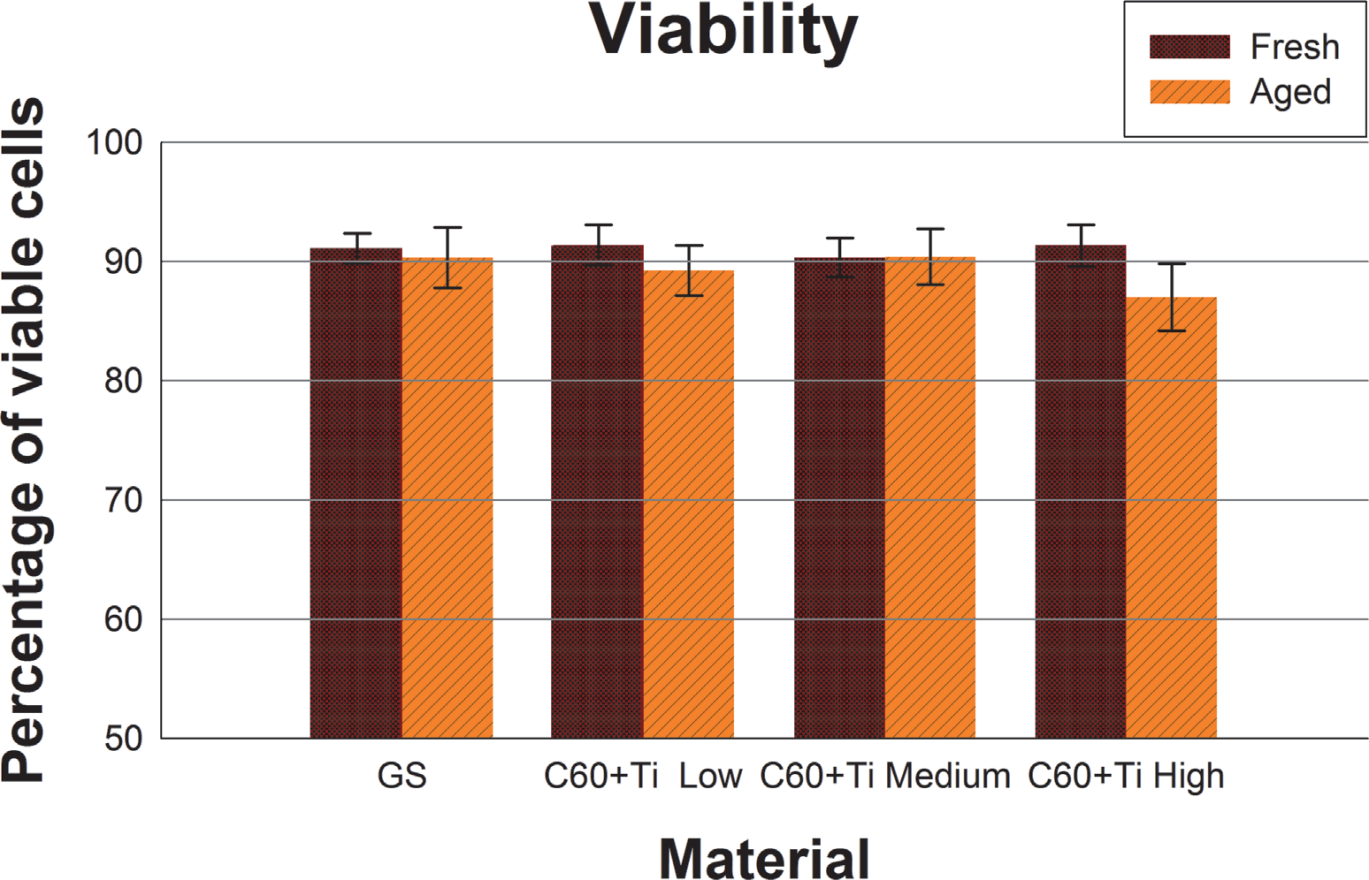
Viability of human osteoblast-like MG-63 cells, measured by the trypan blue exclusion test on day 7 after seeding on fresh and aged C_60_/Ti composites with various Ti concentrations (low: 25%, medium: 45%, high: 70%). GS: microscopic glass coverslips, reference material. No significant differences among the experimental groups were found.

Cell viability and potential cell membrane damage were analyzed by trypan blue staining. The cells growing on all tested C_60_/Ti composites were highly viable (over 80%) and were comparable to the cells on the reference material. No statistically significant differences in viability were observed among the various Ti concentrations or ages of the C_60_/Ti composites (**Fig 9, S5 Table, S1 Dataset**). The improvement in metabolic activity and also in the viability of cells cultured on C_60_/Ti layers (especially fresh composites) is obvious when compared to our previous study performed on pure C_60_ layers [38].

### DNA damage response

It has been reported that fullerenes are able to bind directly to the minor and major grooves of double-strand DNA and to form a stable complex, which may have a negative impact on the self-repairing process of the dsDNA and may lead to a potential cytotoxic effect of fullerenes [42, 43].

**Fig 10:**
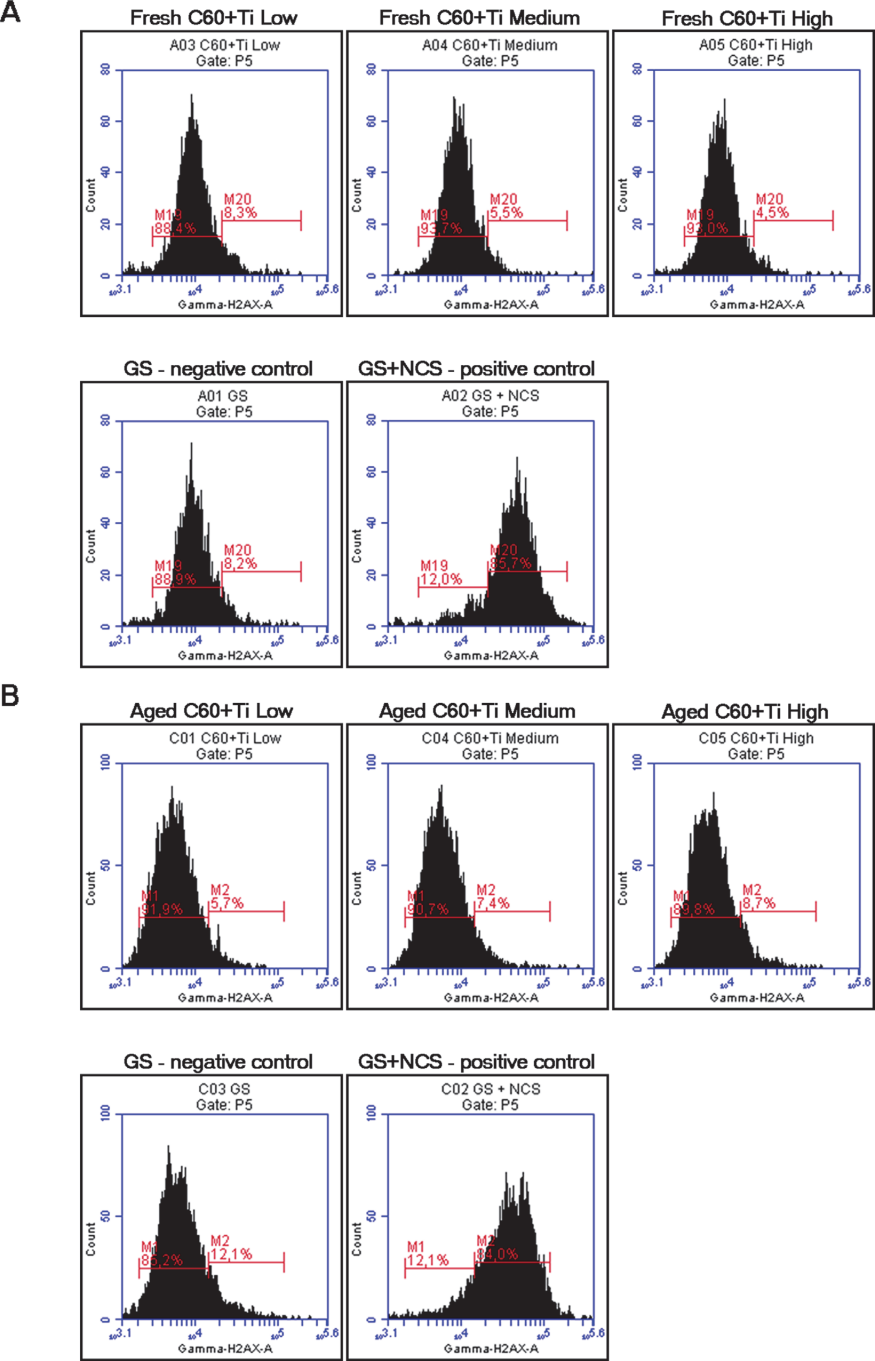
Flow cytometry of the marker of DNA damage response: gamma-H2AX in human osteoblast-like U-2 OS cells on fresh (A) and aged (B) C_60_/Ti composites with various Ti concentrations (low: 25%, medium: 45%, high: 70%). GS: microscopic glass coverslips, reference material; GS+NCS: positive control to phosphorylation of histon H2AX (gamma-H2AX), induced by 1 hour incubation of U-2 OS in neocarzinostatin (NCS; 700ng/mL). M19 and M1 define the percentage of cells with no increase in DNA damage (obtained from cells growing on the reference material, GS); M20 and M2 define the percentage of cells with an increased DNA damage response represented by enhanced phosphorylation of histon H2AX (obtained from cells incubated with NCS).

**Fig 11:**
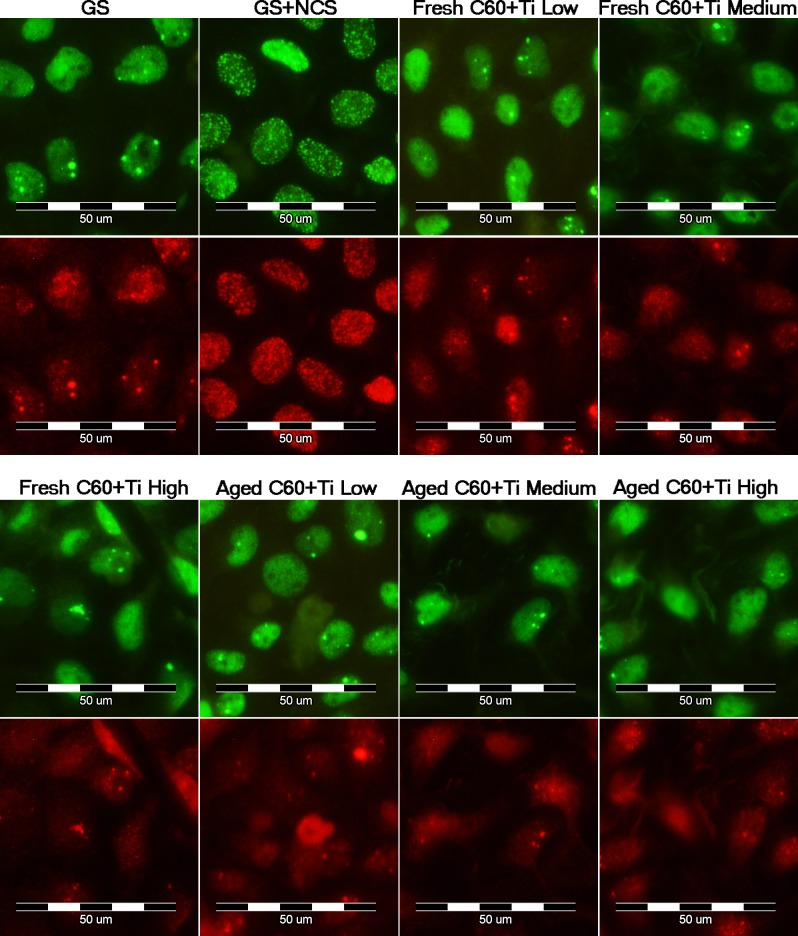
Immunofluorescence staining of markers of a DNA damage response: 53BP1 (green) and gamma-H2AX (red) in human osteoblast-like U-2 OS cells on fresh and aged C_60_/Ti composites with various Ti concentrations (low: 25%, medium: 45%, high: 70%). GS: microscopic glass coverslips, reference material; GS+NCS: positive control of DNA damage response induced by 1 hour incubation of U-2 OS in neocarzinostatin (NCS; 700ng/mL).

We therefore studied the DNA damage response (DDR) of cells growing on fullerene films, by markers of DNA double strand breaks. For this purpose, osteosarcoma cell line U-2 OS was used instead of MG-63, which is p53-deficient. Gamma-HA2X (phosphorylated histon H2AX, a marker of early DDR) and 53BP1 (p53 binding protein), whose focal recruitment depends on a number of upstream factors, were evaluated. After 3 and 7 days of cultivation on various Ti concentrations of fresh and aged C_60_/Ti composites, the level of gamma-H2AX phosphorylation was analyzed by flow cytometry. The results show no increase in the percentage of cells with enhanced phosphorylation of histon H2AX cultured on layers with various Ti additions in comparison to the reference glass coverslips. Moreover, there was no effect of the age of the C_60_/Ti composites on DDR (**Fig 10**). Furthermore, the visualization of both DDR markers by immunofluorescence staining also revealed no increased recruitment or formation of either gamma-H2AX or 53BP1 foci (**Fig 11**). These results are consistent with our previous study focused on C_60_ layers [38]. In accordance with our results, fullerene C_60_ nanoparticles in suspension had no genotoxic ability in the bacterial reverse mutation assay, in the *in vitro* chromosome aberration assay, or in the *in vivo* micronucleus assay [44]. In addition, fullerenol mediated a decrease in the frequency of micronuclei and chromosome aberrations [45].

## Conclusions and Further Perspectives

Our study has revealed that both fresh and aged C_60_/Ti composites are suitable substrates for the adhesion and growth of human bone cells. However, in the case of pure fullerene C_60_ films studied earlier, aged films were better for cell colonization than fresh films, which had a certain negative impact on the cell spreading, proliferation, viability and activity of mitochondrial enzymes. Interestingly, the examination of C_60_/Ti composites in this study showed no significant differences between fresh and aged films (caused by the improvement in the properties of the fresh layers). This difference between pure fullerene films and C_60_/Ti composites may lie in the fact that in the composites, changes in the fullerene molecules, such as fragmentation, polymerization, oxidation and graphitization, occur not only due to aging of the material, but immediately during C_60_ and Ti co-deposition due to the interaction of C_60_ molecules and Ti atoms. In addition, studies performed on human osteoblast-like U-2 OS cells revealed no DNA damage response of these cells cultivated on fresh or aged C_60_/Ti composites. C_60_/Ti composites can therefore be considered as promising materials in bone tissue engineering, namely for potential coating of bone implants. The connection (association) of C_60_ with Ti may also have promising therapeutic potential against oxidative stress-associated conditions and in the treatment of bone and cartilage tissue destruction in arthritis.

## Supporting Information

S1 Dataset Excel sheet of raw data numbers from which the qualitative data, mean ± standard error of the mean (S.E.M) or median with interquartile range (IQR) were calculated. (XLS)

S1 Fig Doubling times (in hours) of human osteoblast-like MG-63 cells cultured on fresh or aged C_60_/Ti composites with various Ti concentrations (low: 25%, medium: 45%, high: 70%). GS: microscopic glass coverslips, reference material. The data from different time intervals (day 1–3 **(A)**, day 3–7 **(B)**) is presented as median with interquartile range (IQR = Q3—Q1) obtained from 3 experiments. No significant differences among the experimental groups were found.
(TIF)

S2 Fig Summarized doubling time (in hours) of human osteoblast-like MG-63 cells cultured on fresh or aged C_60_/Ti composites with various Ti concentrations (low: 25%, medium: 45%, high: 70%). GS: microscopic glass coverslips, reference material. The data is presented as median with interquartile range (IQR = Q3—Q1) obtained from 3 experiments. No significant differences among the experimental groups were found.
(TIF)

S1 Table Static water drop contact angle of fresh and aged C_60_/Ti composites with various Ti concentrations (low: 25%, medium: 45%, high: 70%). The data is presented as mean ± standard error of the mean (S.E.M.) obtained from 10 measurements. *^Aged^ significant difference between fresh and aged layers; ^**+** Low, High^ significant difference to low and high concentration of Ti among the aged samples; p ≤ 0.05.
(DOC)

S2 Table Numbers of human osteoblast-like MG-63 cells on fresh or aged C_60_/Ti composites with various Ti concentrations (low: 25%, medium: 45%, high: 70%) on day 1 (A), 3 (B) and 7 (C) after seeding. The data is presented as mean ± standard error of the mean (S.E.M.) obtained from 3 experiments. GS: microscopic glass coverslips, a reference material. No significant differences among the experimental groups were found.
(DOC)

S3 Table Doubling times (in hours) of human osteoblast-like MG-63 cells cultured on fresh or aged C_60_/Ti composites with various Ti concentrations (low: 25%, medium: 45%, high: 70%). GS: microscopic glass coverslips, reference material. The data from different time intervals (day 1–3 **(A)**, day 3–7 **(B),** and summarized day 1–7 **(C)**) is presented as median with interquartile range (IQR = Q3—Q1) obtained from 3 experiments. No significant differences among the experimental groups were found.
(DOC)

S4 Table Metabolic activity measured by the XTT test per culture of human osteoblast-like MG-63 cells on day 7 after seeding on fresh and aged C_60_/Ti composites with various Ti concentrations (low: 25%, medium: 45%, high: 70%). The data is presented as mean ± standard error of the mean (S.E.M.) obtained from 3 experiments. GS: microscopic glass coverslips, a reference material. No significant differences among the experimental groups were found.
(DOC)

S5 Table Percentage of viable cells (human osteoblast-like MG-63 cells), measured by the trypan blue exclusion test on day 7 after seeding on fresh and aged C_60_/Ti composites with various Ti concentrations (low: 25%, medium: 45%, high: 70%). The data is presented as mean ± standard error of the mean (S.E.M.) obtained from 3 experiments. GS: microscopic glass coverslips, reference material. No significant differences among the experimental groups were found.
(DOC)
