# Supplementary figures and images for: Growth and Potential Damage of Human Bone-Derived Cells Cultured on Fresh and Aged C60/Ti Films
Source: PLoS One. 2015 Apr 15;10(4):e0123680. doi: 10.1371/journal.pone.0123680 (PMC4398559; doi:10.1371/journal.pone.0123680)

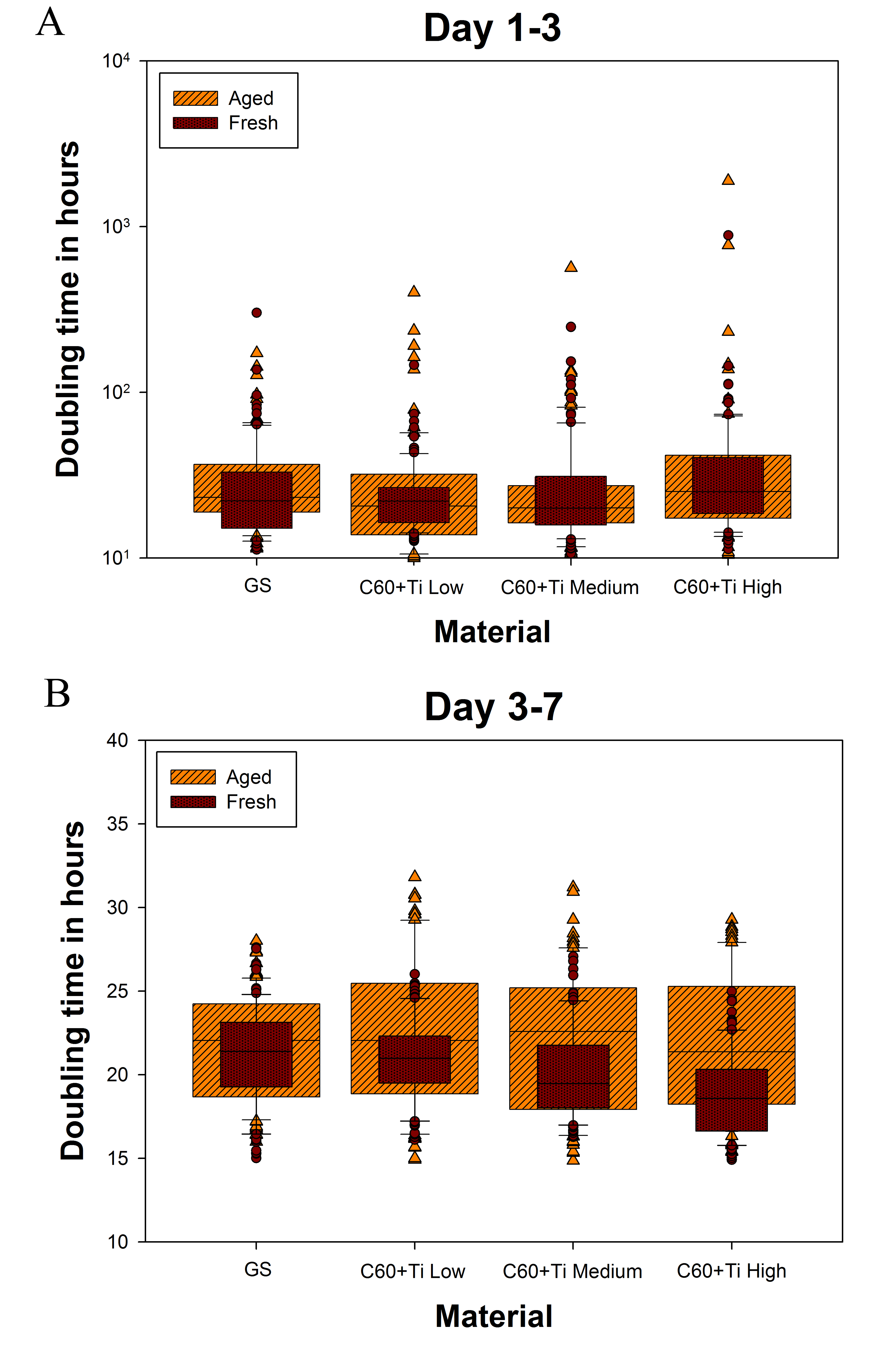

Supplement: S1 Fig — GS: microscopic glass coverslips, reference material. The data from different time intervals (day 1–3 (A), day 3–7 (B)) is presented as median with interquartile range (IQR = Q3—Q1) obtained from 3 experiments. No significant differences among the experimental groups were found. (TIF) [file pone.0123680.s002.tif]

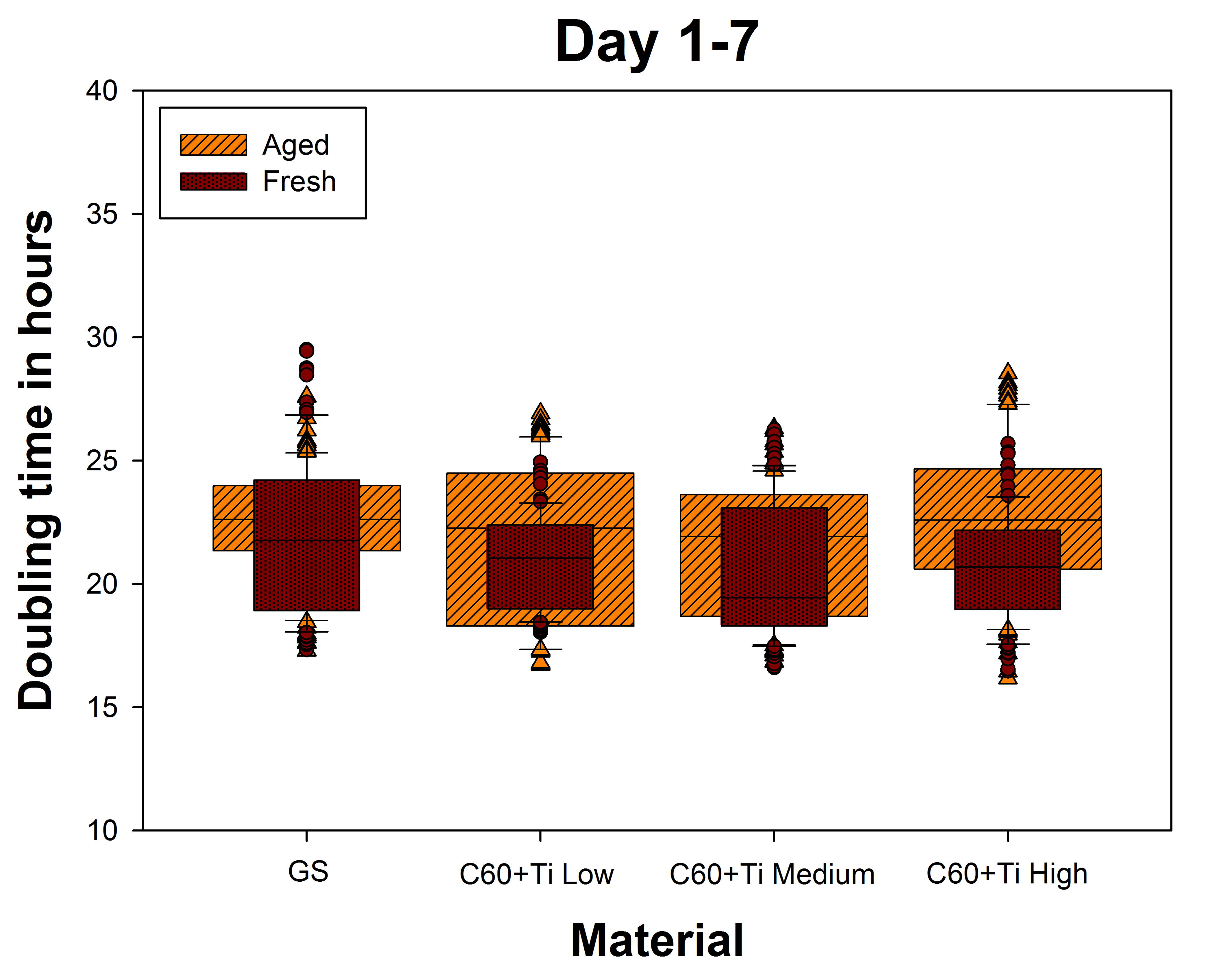

Supplement: S2 Fig — GS: microscopic glass coverslips, reference material. The data is presented as median with interquartile range (IQR = Q3—Q1) obtained from 3 experiments. No significant differences among the experimental groups were found. (TIF) [file pone.0123680.s003.tif]
