# Supplementary material for: Growth and Potential Damage of Human Bone-Derived Cells Cultured on Fresh and Aged C60/Ti Films
Source: PLoS One. 2015 Apr 15;10(4):e0123680. doi: 10.1371/journal.pone.0123680 (PMC4398559; doi:10.1371/journal.pone.0123680)
Supplement: S1 Table — The data is presented as mean ± standard error of the mean (S.E.M.) obtained from 10 measurements. *Aged significant difference between fresh and aged layers; + Low, High significant difference to low and high concentration of Ti among the aged samples; p ≤ 0.05. (DOC) [file pone.0123680.s004.doc]

**Tab S1.** Static water drop contact angle of fresh and aged C_60_/Ti composites with various Ti concentrations (low: 25%, medium: 45%, high: 70%). The data is presented as mean ± standard error of the mean (S.E.M.) obtained from 10 measurements. *^Aged^ significant difference between fresh and aged layers; **^+^** ^Low, High^ significant difference to low and high concentration of Ti among the aged samples; p ≤ 0.05.

| **Contact angle** | **Fresh** | **Aged** |
| --- | --- | --- |
| **Samples** | **Mean±SEM** | **Mean±SEM** |
| C_60_/Ti Low | 98.4 ± 0.8*^Aged^ | 94.8 ± 0.9 |
| C_60_/Ti Medium | 97.4 ± 0.8*^Aged^ | 89.6 ± 1.9 **^+^** ^Low, High^ |
| C_60_/Ti High | 97.8 ± 1.0 | 97.0 ± 1.0 |
