# Supplementary material for: Growth and Potential Damage of Human Bone-Derived Cells Cultured on Fresh and Aged C60/Ti Films
Source: PLoS One. 2015 Apr 15;10(4):e0123680. doi: 10.1371/journal.pone.0123680 (PMC4398559; doi:10.1371/journal.pone.0123680)
Supplement: S2 Table — The data is presented as mean ± standard error of the mean (S.E.M.) obtained from 3 experiments. GS: microscopic glass coverslips, a reference material. No significant differences among the experimental groups were found. (DOC) [file pone.0123680.s005.doc]

**Tab S2.** Numbers of human osteoblast-like MG-63 cells on fresh or aged C_60_/Ti composites with various Ti concentrations (low: 25%, medium: 45%, high: 70%) on day 1 **(A)**, 3 **(B)** and 7 **(C)** after seeding. The data is presented as mean ± standard error of the mean (S.E.M.) obtained from 3 experiments. GS: microscopic glass coverslips, a reference material. No significant differences among the experimental groups were found.

**A**

| **Day 1** | **Fresh** | **Aged** |
| --- | --- | --- |
| **Samples** | **Mean±SEM** | **Mean±SEM** |
| GS | 2,579 ± 572 | 2,619 ± 363 |
| C_60_/Ti Low | 1,919 ± 274 | 2,101 ± 405 |
| C_60_/Ti Medium | 1,716 ± 384 | 2,192 ± 357 |
| C_60_/Ti High | 1,722 ± 291 | 2,382 ± 415 |

| **Day 3** | **Fresh** | **Aged** |
| --- | --- | --- |
| **Samples** | **Mean±SEM** | **Mean±SEM** |
| GS | 9,560 ± 1,291 | 10,339 ± 1,405 |
| C_60_/Ti Low | 9,165 ± 1,087 | 9,940 ± 1,792 |
| C_60_/Ti Medium | 7,533 ± 1,121 | 10,965 ± 1,794 |
| C_60_/Ti High | 6,099 ± 967 | 8,874 ± 1,502 |

**B**

| **Day 7** | **Fresh** | **Aged** |
| --- | --- | --- |
| **Samples** | **Mean±SEM** | **Mean±SEM** |
| GS | 218,263 ± 10,663 | 217,381 ± 10,384 |
| C_60_/Ti Low | 221,502 ± 9,402 | 188,215 ± 12,825 |
| C_60_/Ti Medium | 205,567 ± 11,166 | 222,204 ± 17,388 |
| C_60_/Ti High | 206,273 ± 11,509 | 190,573 ± 18,593 |

**C**
