# Supplementary material for: Growth and Potential Damage of Human Bone-Derived Cells Cultured on Fresh and Aged C60/Ti Films
Source: PLoS One. 2015 Apr 15;10(4):e0123680. doi: 10.1371/journal.pone.0123680 (PMC4398559; doi:10.1371/journal.pone.0123680)
Supplement: S3 Table — GS: microscopic glass coverslips, reference material. The data from different time intervals (day 1–3 (A), day 3–7 (B), and summarized day 1–7 (C)) is presented as median with interquartile range (IQR = Q3—Q1) obtained from 3 experiments. No significant differences among the experimental groups were found. (DOC) [file pone.0123680.s006.doc]

**Tab S5.** Doubling times (in hours) of human osteoblast-like MG-63 cells cultured on fresh or aged C_60_/Ti composites with various Ti concentrations (low: 25%, medium: 45%, high: 70%). GS: microscopic glass coverslips, reference material. The data from different time intervals (day 1-3 **(A)**, day 3-7 **(B),** and summarized day 1-7 **(C)**) is presented as median with interquartile range (IQR = Q3 – Q1) obtained from 3 experiments. No significant differences among the experimental groups were found.

**A**

**B**

**C**
