# Supplementary material for: Growth and Potential Damage of Human Bone-Derived Cells Cultured on Fresh and Aged C60/Ti Films
Source: PLoS One. 2015 Apr 15;10(4):e0123680. doi: 10.1371/journal.pone.0123680 (PMC4398559; doi:10.1371/journal.pone.0123680)
Supplement: S4 Table — The data is presented as mean ± standard error of the mean (S.E.M.) obtained from 3 experiments. GS: microscopic glass coverslips, a reference material. No significant differences among the experimental groups were found. (DOC) [file pone.0123680.s007.doc]

**Tab S6.** Metabolic activity measured by the XTT test per culture of human osteoblast-like MG-63 cells on day 7 after seeding on fresh and aged C_60_/Ti composites with various Ti concentrations (low: 25%, medium: 45%, high: 70%). The data is presented as mean ± standard error of the mean (S.E.M.) obtained from 3 experiments. GS: microscopic glass coverslips, a reference material. No significant differences among the experimental groups were found.

| **Absorbance** | **Fresh** | **Aged** |
| --- | --- | --- |
| **Samples** | **Mean±SEM** | **Mean±SEM** |
| GS | 0.53± 0.02 | 0.54 ± 0.03 |
| C_60_/Ti Low | 0.51 ± 0.03 | 0.45 ± 0.04 |
| C_60_/Ti Medium | 0.49 ± 0.01 | 0.52 ± 0.03 |
| C_60_/Ti High | 0.50 ± 0.03 | 0.48 ± 0.04 |
