# Supplementary material for: Growth and Potential Damage of Human Bone-Derived Cells Cultured on Fresh and Aged C60/Ti Films
Source: PLoS One. 2015 Apr 15;10(4):e0123680. doi: 10.1371/journal.pone.0123680 (PMC4398559; doi:10.1371/journal.pone.0123680)
Supplement: S5 Table — The data is presented as mean ± standard error of the mean (S.E.M.) obtained from 3 experiments. GS: microscopic glass coverslips, reference material. No significant differences among the experimental groups were found. (DOC) [file pone.0123680.s008.doc]

**Tab S7.** Percentage of viable cells (human osteoblast-like MG-63 cells), measured by the trypan blue exclusion test on day 7 after seeding on fresh and aged C_60_/Ti composites with various Ti concentrations (low: 25%, medium: 45%, high: 70%). The data is presented as mean ± standard error of the mean (S.E.M.) obtained from 3 experiments. GS: microscopic glass coverslips, reference material. No significant differences among the experimental groups were found.

| **Viability in %** | **Fresh** | **Aged** |
| --- | --- | --- |
| **Samples** | **Mean±SEM** | **Mean±SEM** |
| GS | 91.1 ± 1.3 | 90.3 ± 2.5 |
| C_60_/Ti Low | 91.4 ± 1.7 | 89.3 ± 2.1 |
| C_60_/Ti Medium | 90.3 ± 1.6 | 90.4 ± 2.3 |
| C_60_/Ti High | 91.3 ± 1.7 | 87.0 ± 2.8 |
